# Deconstructing Wine Grape Cell Walls with Enzymes During Winemaking: New Insights from Glycan Microarray Technology

**DOI:** 10.3390/molecules24010165

**Published:** 2019-01-04

**Authors:** Yu Gao, Anscha J. J. Zietsman, Melané A. Vivier, John P. Moore

**Affiliations:** 1 Center for Viticulture and Enology, Department of Plant Science, School of Agriculture and Biology, Shanghai Jiao Tong University, Shanghai 200024, China; yugao@sjtu.edu.cn; 2 Institute for Wine Biotechnology, Department of Viticulture and Oenology, Faculty of AgriSciences, Stellenbosch University, Matieland 7602, South Africa; jjv2@sun.ac.za (A.J.J.Z.); mav@sun.ac.za (M.A.V.)

**Keywords:** wine maceration, glycomics, cell wall architecture, cell wall-polyphenol interactions, analytical methods

## Abstract

Enzyme-aid maceration is carried out in most modern winemaking industries with a range of positive impacts on wine production. However, inconsistencies in enzyme efficiency are an issue complicated by unclear targets (limited information available on berry cell wall architecture of different cultivars) and the complex wine environment (i.e., fermenting must). Recent studies have been performed to develop a clearer picture of grape cell wall structures, maceration effects, and interactions between important wine compounds and grape-derived polysaccharides. This review highlights critically important recent studies on grape berry cell wall changes during ripening, the importance of enzymes during maceration (skin contact phase) and deconstruction processes that occur during alcoholic fermentation. The novelty of the Comprehensive Microarray Polymer Profiling (CoMPP) technique using cell wall probes (e.g., antibodies) as a method for following cell wall derived polymers during different biological and biotechnological processes is discussed. Recent studies, using CoMPP together with classical analytical methods, confirmed the developmental pattern of berry cell wall changes (at the polymer level) during grape ripening. This innovative technique were also used to track enzyme-assisted depectination of grape skins during wine fermentation and determine how this influence the release of wine favourable compounds. Furthermore, polysaccharides (e.g., arabinogalactan proteins) present in the final wine could be identified. Overall, CoMPP provides a much more enriched series of datasets compared to traditional approaches. Novel insights and future studies investigating grape cell wall and polyphenol interactions, and the tailoring of enzyme cocktails for consistent, effective and “customized” winemaking is advanced and discussed.

## 1. Introduction

**Figure 1:**
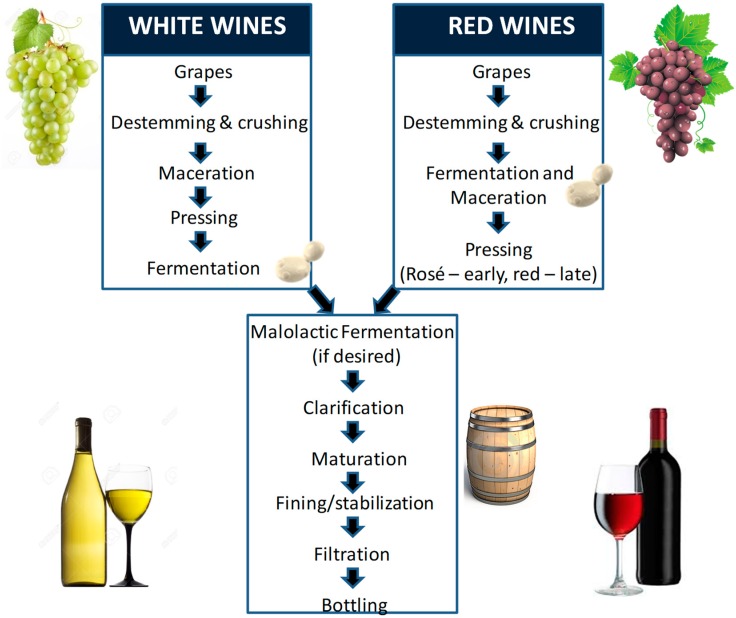
An example of a winemaking process for white and red wines. In this case maceration was included in the white winemaking process, this is optional, and not a common practice.

Winemaking is an ancient art and a science having been performed for thousands of years with a range of practices-interventions (e.g., such vineyard management, harvest methods, maceration techniques, etc.) developed and crucial in order to take the grapes from the vine to the final wine [1]. Outlines of the standard winemaking processes for white and red wines are provided (see Figure 1). These procedures are refined as new knowledge is applied to the viticultural (i.e., science of grapevine and grape growing) and oenological (i.e., science of wine and winemaking) methods available to the global wine industry. Additionally, more fundamental knowledge from a molecular, genetics and physiological perspective of grapevine and wine organisms (yeast and bacteria) have ensured that the wine industry is at the forefront of scientific research and innovation. Harvested grapes are the start of any winemaking process and here knowledge of starting input material is crucial for successful winemaking and quality wine. To unlock the potential of the grape matrix (e.g., the harvested berries) methods to macerate (i.e., break down) and ferment the berries into final wine are required. Methods to macerate grapes include physical (i.e., mechanical presses) and biochemical (e.g., added enzymes as oenological aids) processes which are needed to break open the grape tissues. Grape berries are composed of tissues and cells which in turn are surrounded by polysaccharide-rich cell wall(s). Grape cell walls need to be deconstructed (broken down) in order to release components such as metabolites (e.g., sugars, acids, volatiles, pigments) and polymers (e.g., pectins, proteins, polyphenols such as tannins) into the fermenting must (i.e., grape juice) for wine production [1]. Adding enzymes from GRAS (generally regarded as safe) fungal sources is usually performed to enhance extraction of pigments and other critical metabolites (e.g., polyphenols) in red winemaking; enzymes also increase juice volume for both red and white winemaking processes; and enhance clarification in white winemaking. A major challenge in determining the suitability of enzymes as interventions has been a general lack of scientific knowledge around grape berry cell wall composition and architecture. 

In general, apart from grapes, studying plant cell walls are challenging for a number of reasons; one major challenge is that plant cell walls are higher order composites of a variety of polymers (e.g., polysaccharides, glycoproteins, polyphenolic polymers). The composition of primary plant cell walls varies between species, organs (e.g., fruits such as grape berries), tissues and individual cells [2]. Furthermore, there is still a lot of debate on the accuracy of the various structural models proposed (e.g., tethered-network, biomechanical hotspots etc.) and the importance of covalent and non-covalent cross-linking between constituent plant cell wall polymers [3,4]. Current plant cell wall models may thus not be useful or accurate with respect to those present in grape berry cell walls.

During the last couple of decades, a number of studies have used classical cell wall analytical methods (such as chemical fractionation) to generate novel and valuable information on grape berry cell wall composition [5,6] and how this polymer composite changes during fruit development (e.g., ripening). From an economic and industry perspective the changing nature (e.g., deconstruction) of grape berry cell walls during wine maceration and clarification have been studied indirectly by looking at several measurable parameters (e.g., tannins, anthocyanins released) that indicate grape cell wall breakdown [7,8]. The effective breaking down of grape berry cell walls is thus important in the context of ‘quality wine’ because of: (1) better and balanced extraction of favorable compounds, (2) higher juice yield and more consistent quality, and (3) enhanced typicity of cultivar wines (e.g., Shiraz wine style) [9]. Enzymes are oenological aids, which help degrade polysaccharide-rich grape berry cell walls leading to enhanced wine composition and consistency [9]. However, as most methods are indirect (e.g., wine colour, juice volume etc.) the role of enzymes in grape cell wall degradation has been difficult to assess. In the last decade novel and innovative cell wall technologies [10], have been developed which has allowed the detection of grape polysaccharides directly, by virtue of their epitopes, and in conjunction with classical methods have permitted new insights for the grape and wine sciences.

## 2. The Cell Wall Composition of the Grape Berry from Structure to Function

**Figure 2:**
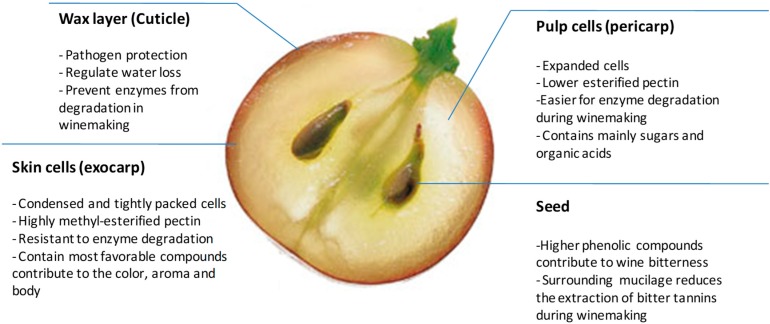
The biological anatomy and biochemical composition of a typical wine grape berry with reference to extractable components.

In general, grape berries are composed of three main tissue types [11]; these being the skins, the pulp and the seeds. The cuticle (Figure 2), that covers the skin, is the primary interface between the plant and the environment and is a protective layer (against pathogens and minimizes water loss) that consists of waxes (soluble lipids) embedded in or deposited on the cutin-rich matrix [12]. In red winemaking, this wax layer most probably, albeit not proven, prevents cell wall degrading enzymes from penetrating into the inner tissues (e.g., skin and pulp), thus enzymes can only penetrate effectively from the pulp exposed during grape crushing. In addition, the cutin-rich skin layer will trap air within the pomace (crushed grapes) causing it to float (forming the cap) on the top of fermenting must (grape juice). Red winemaking requires daily punch-downs of the pomace into the must to assist maceration and minimize the risks of wine spoilage due to oxygen exposure.

Condensed and tightly packed skin cells (i.e., exocarp) are located [5] beneath the cuticular layer, which during ripening accumulate phenolic compounds that when released during maceration positively contribute to wine quality and sensory characteristics [13]. The grape skin constitutes 5–10% of the total dry weight of the grape berry [14]. The number of cell layers in the grape berry skin, the size of the cells and the cell wall composition are cultivar-specific [15]. However, on average the skin cell walls consist of 30% neutral polysaccharides (cellulose, xyloglucan, arabinan, galactan, xylan and mannan) and a further 20% of acidic pectin components (62% methyl esterified) [14] on a weight basis. A more recent iterative calculation method inferred the polysaccharides present in skin cell walls [16]. With this method they estimated the polysaccharides to be 57–62 mol % homogalacturonan (HG), 6–14 mol % cellulose, 10–11 mol % xyloglucan, 7 mol % arabinan, 4.5–5 mol % RG-I, 3.5–4 mol % RG-II, 3 mol % arabinogalactan and 0.5–1.0 mol % mannans.

The pulp (i.e., flesh, also known as pericarp) is the main storage tissue for free sugars (i.e., glucose and fructose) and organic acids (i.e., tartaric acid) [17]. Pulp cells and tissues expand significantly during and after the veraison stage by volume compared to skin cells which expand by net surface area (i.e., a surface-to-volume ratio) [18]. Pulp tissue cell wall layers are comprised mainly of cellulose and pectin polysaccharides in addition to extensin proteins [18]. Ortega-Regules et al. [15] determined the sugar composition of the cell walls from both the skin and the pulp cells of four different wine grape cultivars. Pulp cell walls had higher fucose, rhamnose, and xylose but lower arabinose, galactose, mannose and glucose than the hemicellulose-rich skin cells. Uronic acid levels were similar between the different tissues but the degree of methylesterification was significantly lower in pulp cell walls [19]. Within the pulp tissue are also the grape seeds, which harbor a high concentration of phenolic compounds, compared with the skins and pulp. Most seed phenolics and tannins are very harsh and bitter and are therefore unwanted extractives in winemaking. An outer impermeable layer forms around the seeds as berry ripening proceeds [20] but no cell wall data is currently available on seeds or seed mucilage tissues.

## 3. Grape Cell Wall Integrity Associated with Ripening and Berry Health 

Grape berry ripening consist of a cell division (green) phase followed by a cell expansion (ripe) phase [21]. The onset of this second phase known as veraison is marked by the initiation of events such as sugar accumulation, a decrease in organic acids, colour development, berry expansion and fruit softening. Information regarding fruit ripening or softening is commercially important because it is closely associated with the development of flavor and colour indicators for optimum ripeness [22]. Over-ripening can be deleterious as excessive softening leads to mechanical damage and microbial spoilage of fruit either on the vine or during postharvest handling. Textural changes that occur during ripening partially correlate with cell wall polysaccharide remodeling [23]. Berry ripening links with size and morphological changes and a series of coordinated biochemical processes. Both biosynthetic and degradative metabolism of cell wall components involve numerous plant enzymes. Several reviews [24,25,26] discuss in detail the processes and the enzymes involved in plant cell wall turnover.

Nunan et al. studied grape berry cell walls at different developmental stages using light and fluorescence microscopy. The microscopy study revealed limited information and no differences in grape tissue cell wall thickness [5]. A further compositional analysis showed a major decrease in polymeric galactose (e.g., galactans) content during ripening of the berry, especially the (1,4)-linked galactopyranose residues of type I arabinogalactan [27]. The polymeric galactose decrease correlates with the grape β-galactosidase activity that is present in all stages of ripening berries [27]. Furthermore, grape β-galactosidase encoding mRNA accumulates from pre-veraison until early post-veraison in berries [27]. During ripening there is also a marked increase in the solubility of pectic polysaccharides [5] which correlates with α-galactosidase, β-galactosidase and pectin methylesterase activities measured in grapes [27]. Pectin solubilisation through action of polygalacturonases and/or pectate lyases is supported by the corresponding genes, which are actively expressed post-veraison. Grapes, in contrast to other fruits, show only a slight decrease (from 58 to 48%) in the degree of methyl esterification in the skin cell walls during ripening. This is further supported by epitope data for highly esterified HG (homogalacturonan) versus lower levels of esterified HG in ripe Cabernet Sauvignon grape pomace [28]. An increase in cell wall associated proteins, particularly hydroxyproline-rich proteins, was inferred from changes in amino acid profiles, as well as epitope abundance data [21], whereas cellulose and xyloglucan levels remained unchanged. 

A number of studies have suggested that berry cell walls show cultivar specific differences during development and ripening. Yakushiji and co-workers [29] for example studied the skin cell walls of *Vitis vinifera* L. × *Vitis labrusca* L. during veraison and found, in contrast to Nunan [5], that the cellulose contents actually decreased. They also noted a depolymerization of xyloglucan and pectic polysaccharides and a general decrease in hemicellulose polymers of ripening grapes [29]. Guillaumie et al. (2011) for example, demonstrated that the expression profiles of four xyloglucan endotransglycosylases/hydrolyse encoding genes followed a ripening pattern in Chardonnay fruit. Whereas Moore et al. showed a decrease in epitope abundance for mAb LM15 (a probe that binds to unsubstituted xyloglucan) with ripening in Cabernet Sauvignon [21,30]. Ortega-Regules and co-workers [6] investigated the skin cells from veraison to technological maturity (an industry term related to the sugar concentration, titratable acidity and pH levels of the grape juice), and showed with transmission microscopy that the cell walls become progressively thinner as the grapes ripen [6]. This correlates with a decrease in cell wall material per gram of skin as ripening progressed. Mourvèdre (Monastrell), Merlot and Cabernet Sauvignon cultivars showed a decrease in polymeric galactose levels in pulp (similar to skin cell walls) and a decrease in pectin methyesterification and acetylation levels whereas Shiraz did not show any of these changes. Cultivar specific differences therefore have downstream effects on the subsequent winemaking processes and require careful consideration. 

In addition to the effect of endogenous processes on grape berry cell wall integrity, extrinsic factors are also important. The quality of harvested grape berries is crucial for successful winemaking. Spoilage bacteria and fungi that colonise grape berries pre- and post-harvest produce cell wall degrading enzymes that rot the fruit before wine can be made resulting in significant losses for the industry [31]. Necrotrophs (such as *Botrytis cinerea* the grey rot fungus) and biotrophs (such as downey and powdery mildew fungi) produce cell wall degrading enzymes during fruit colonization. The genome sequence of *Botrytis cinerea* reveals an arsenal of cell wall degrading enzymes [32]. Some of these enzymes (functional annotations) facilitate backbone deconstruction (e.g., endo-polygalacturonase, pectin lyase, pectin methyl-esterase), and side chain cleavage (e.g., arabinase) [32]. The infected grapes detect the pathogen and mount a defense response [33,34]. Oligosaccharides released from HG backbone of the grape cell wall can act as potent defense response elicitors and activate plant immune responses [35,36,37]. Upregulation of endogenous grape PME (pectin methyl-esterase) activity is believed to increase the levels of cell wall de-esterified HG (homogalacturonans) facilitating the production of free oligosaccharides [38]. Polygalacturonase inhibiting proteins (PGIPs) (an example of pathogen related cell wall proteins) are produced in grape berry tissues where they are believed to modulate defense responses in grapevine in response to pathogenic fungal infection [39,40]. All of these defense responses take place in the plant cell wall matrix where they influence directly or indirectly on winemaking processes and wine quality. 

Finally, vineyard and environmental factors play a crucial role in grape health and maturity. Here the ‘terroir’ affect is often considered when comparing vineyards between each other but intra-vineyard variability is also of great importance. This is evident by measuring several parameters such as sugars, organics acids and anthocyanins amongst others [41,42]. A recent study by Gao et al. used glycan microarray technology to evaluate berry ripening status with cell wall composition within a Cabernet Sauvignon vineyard [19]. The study showed that intra-vineyard variation at harvest is brought into the winemaking process and has the potential to result in incomplete berry fruit cell wall degradation and inconsistencies in the resultant wine quality parameters (e.g., phenolics). Therefore, grape cell wall integrity (in addition to ripening indicators such as sugars and acids) require measurement (i.e., methods developed) and consideration when choosing to harvest grapes for winemaking.

## 4. The Benefits and Drawbacks of Maceration in Winemaking

Harvested grapes are the starting material for winemaking; however, grape batches are inherently variable with respect to quality parameters. Grape tissues contain sugars and acids in the more easily disrupted pulp cells whereas the skin cells contain the pigments, flavor compounds and phenolic compounds (e.g., tannins) necessary for quality wine production. The condensed tightly packed skin cells are very resistant to the mechanical disruption (crushing steps), which makes the release of favorable chemical compounds (e.g., polyphenols) difficult [11]. Thus, one of the most important objectives in winemaking is to enhance the effective breakdown of the berry cell wall during the skin contact phase [9].

A number of winemaking techniques have been developed for more effective extraction and maceration. Cold maceration involves a short pre-fermentation period at 4 to 15 °C before the standard fermentation commences. This pre-fermentation assists in the release of more compounds that are favorable during the subsequent standard winemaking steps [43]. Thermovinification involves applying elevated temperatures as well as the extended maceration times to the fermentation stages in order to enhance phenolic extraction through heat release [44]. Physical disruption such as electric pulse treatments are effective for leaching of polyphenols and flavor compounds from grape tissues [45]. A major challenge with these physicochemical methods is a lack-of-consistency and unwanted side-effects such as the formation of volatile acidity and off-flavors due to spoilage [46].

Commercial enzyme preparations have been used for many decades in the winemaking industries. These enzymes are crude extracts and are mainly sourced from *Aspergillus* species (such as *Aspergillus niger*) which are GRAS (generally regarded as safe) a recognized status by the OIV (Organisation International de la Vigne et du Vin) (http://www.oiv.int/oiv/info/enspecificationproduit). The main applications of wine enzymes are to increase free run juice volumes, improve or ensure wine clarification and enhance pigment and tannin extractability [47]. These enzyme preparations accelerate maceration and enhance compound extraction into the final wine produced [47,48]. The general belief is that these commercial enzymes act by cleaving skin cell wall polysaccharides releasing polyphenolic tannins and anthocyanins during wine fermentation [7]. Red winemaking requires release of these polyphenols for stable colour, good body and structure and ageing potential of wines [8]. A range of studies over the last 40 years has investigated how maceration enzymes act and influence wine properties [8,11,19,47,48,49,50,51,52,53]. A major reason for the continued interest in the subject from a commercial and scientific viewpoint is that the results achieved by using enzymes for wine preparation are not reproducible between seasons (vintages), between grape cultivars (e.g., Monastrell versus Cabernet Sauvignon) and show batch-to-batch variability. Some studies have suggested and demonstrated that enzymes have very little or no observable/detectable influence on wine properties [48,54,55]. Other studies suggested some effects on wine properties but observed that they were not long lasting [7,56]. Commercial enzyme preparations vary between suppliers and sometimes lack the optimal enzyme activity profiles need for the applications being carried out [53,57]. Of serious concern is the presence of unwanted side or contaminating enzyme activities in commercial products [52,57].

A survey of a number of commercial enzyme preparations for winemaking revealed the presence of polygalacturonase-rich pectinase activities amongst other detected activities [58]. It was predicted that the combination of enzyme activities present in the crude fungal (pectinase) semi-purified preparations would be sufficient for cell wall dissolution; however, these mixtures were inconsistent in their effects [49,59,60]. Another disadvantage with using ineffective enzymes is that discarded fermented pomace contains a large residual amount of phenolic compounds, which needs bioremediation and bio-valorization for appropriate disposal. A major challenge in attempting to develop and assess commercial enzyme mixtures for wine maceration is that very little knowledge on the actual polymeric composition and architecture of grape cell walls was until recently available.

## 5. Polyphenol Extractability during Wine Fermentation, Interaction with Cell Wall Polymers and the Role of Maceration Enzymes 

### 5.1. Extractability of Polyphenols from the Grape Berries during Crushing and Their Interactions with Cell Walls during Winemaking 

Quality wines usually contain high levels of non-volatile phenolic compounds [61] which are important contributors towards the flavour, colour, mouth-feel (astringency and bitterness) and the health promoting properties (anti-oxidants, anti-inflammatory etc.) of wine. The major phenolic compounds found in grapes and wines are the hydroxycinnamic acids, stilbenes, anthocyanins and tannins [13]. The polyphenols are mainly compartmentalized in the cell vacuoles with a smaller fraction being bound to the cell wall layers. The polyphenols are released when the grapes cells are physically disrupted (grape crushing) or the cell walls degraded during maceration but even with extended maceration only a fraction of the total polyphenols present in the grapes, will be extracted [11]. The extraction of polyphenols from the grape berry into the wine is in essence a diffusion process and thus depends on the molecular size and the type of polyphenol, the time and temperature of the extraction process, the concentration gradient, cell permeability, the composition of the extraction medium (for example ethanol concentration) and the surface area over the concentration gradient. Once polyphenolic compounds are released during wine processing they can form hydrogen bonds and hydrophobic interactions with the hydroxyl groups and the aromatic and glycosidic oxygen atoms of the cell wall polysaccharides that are present in the wine or those that are still integrated in the cell walls of the grape pomace [62]. Most research on polyphenol and grape cell wall interactions were performed on tannins with a lesser degree on anthocyanins.

Rustioni et al. reported that about 30 % of tannins are non-covalently bonded and another 7–18% (cultivar dependent) is covalently bonded [63]. The two major classes of tannin-binding macromolecules are pectins and proteins (proline rich and/or hydrophobic) [64]. There are some indications that grape cultivars with a high protein or pectin content in their cell walls have a greater affinity for tannins [65,66,67]. However, the strong interaction between Monastrell grape cell walls and tannins [65,66] was not only ascribed to the high pectin content of this cultivar, but also the nature of their cell wall surface conformations. The strength of the interaction between cell wall polysaccharides and tannins increase as the degree of polymerization of the tannins increase (example can be found in apple tissue) and tannins have a strong affinity especially for highly methylated (DM 72%) pectins [68]. Similar results have also been found with anthocyanins and pectins [69]. The different cell wall polysaccharides show differences in their affinity to bind proanthocyanidins. The strongest affinity is between proanthocyanidins and pectins, and then xyloglucan, followed by starch and then lignocellulose. This hierarchy in affinity is a consequence of the three dimensional structures of the respective molecules involved which can prevent or promote access of the proanthocyanidins to the binding sites on the polysaccharides [70]. Riou et al. (2002) also showed that it was specifically RG-II dimers and not monomers that formed complexes with tannins [71]. Grape pulp cell walls showed a higher binding capacity for tannins than skin cell walls [50,72,73]. However, this might only be due to the more effective removal of tannins from flesh cells than from skin cells during the cell wall preparation step prior to these experiments on binding capacity. 

Another factor that influences the interaction between cell wall components and tannins is the ripeness levels of the grapes. The tannin binding capacity increases as the grape berries ripen [11,70,73]. This is quite ironic taking into account that the extractability of phenolic compounds generally increase with the ripeness levels of grapes, as discussed earlier. The higher protein content of ripe grape skins may contribute to the higher adsorption of proanthocyanidins compared to veraison skins cell walls [50,74]. Furthermore, the unripe skins contain more lignin and the resulting higher structural rigidity will impact negatively on cell wall interaction with high molecular mass proanthocyanidins. Bindon et al. speculated that the increase in the binding of specifically high molecular mass tannins is linked to the increase in cell wall porosity (surface area) that happens during ripening and leads to more effective penetration and encapsulation of tannins. The levels of most phenolic compounds in grape tissue increase during ripening followed by a slight decline close to, what can be considered as, the overripe stage [75]. The decline is due to their degradation, their bioconversion into other products or their covalent association with other cellular components. Similarly, anthocyanin biosynthesis that starts during veraison, accumulates in the skin cells during ripening and shows a slight decrease in concentration in overripe grape skins [75,76]. In contrast to proanthocyanidins only a few studies were performed on anthocyanin interactions with grape cell wall polysaccharides. Anthocyanins are located in the vacuole and only come into contact with cell wall polymers during processing [69]. The presence of anthocyanins improves the extraction of tannins from both skin and seeds and anthocyanins compete with tannins for cell wall adsorption sites [77]. The opposite is not true: The level of tannin in wine/solution does not influence the level of anthocyanin extractability [65]. In general, anthocyanins from the skin are extracted early in the maceration while the tannins are extracted later [78], which seems to be linked to the solubility characteristics of these molecules [48].

The ease of extractability of polyphenols from berry skin differs between cultivars [79] and Monastrell (also known as Mourvèdre) have a high extractability index (not easy to extract) [80] compared to cultivars such as Merlot, Shiraz and Cabernet Sauvignon. Ortega-Regules and co-workers [81] found that a low extraction index (easy to extract) was linked to a low concentration of galactose in the cell wall. In addition, a low degree of pectin methylation and acetylation are typical characteristics of more ripe berries [82,83]. Studies have also found links between cell wall sugar composition, grape ripeness levels and the corresponding ease of phenolic extractability [83,84,85]. Grape cell wall data appears to correlate well with ripeness, colour, phenolic composition, and polysaccharide concentrations in Cabernet Sauvignon and Tempranillo final wines. Furthermore, they found that mature grapes released more proanthocyanidins from skins than from seeds. This altered proanthocyanidin levels seemed to contribute positively towards the sensory attributes of the wine (i.e., lower astringency and bitterness). However, with extended maceration the anthocyanin concentration decreased while the corresponding polysaccharide and proanthocyanidin concentration increased. The galloylation percentage of these tannins also increased, suggesting proanthocyanidin extraction from seeds.

### 5.2. The Effect of Maceration Enzymes on the Polyphenol-Cell Wall Polysaccharide Interactions

Traditionally maceration enzymes were believed to partially degrade the grape cell walls which would result in the release of more polyphenolic compounds and an increase in wine quality. Recent studies that highlight the affinity between cell wall polysaccharides and these polyphenolic molecules, emphasise the need to better understand the influence that maceration enzymes have on not only the release, but also on the interaction between cell wall polysaccharides and polyphenolic compounds.

Due to the complexity of the wine matrix most studies that investigate these issues use model systems; e.g., isolated cell walls and isolated tannins react in a model wine solution (typically 12% ethanol, pH 3.6). Unfortunately these conditions do not render a true reflection of the dynamic environment of a macerating must where a number of processes takes place: e.g., continuous depolymerisation of cell wall polysaccharides, release of oligosaccharides and the liberation of polyphenols, the simultaneous formation of new complexes between different polyphenols and between polyphenols and cell wall components. All of these interactions are also modulated by the ever changing environment (e.g., increase in alcohol, decrease in fermenting sugars). Nevertheless, from these studies it was seen that a commercial pectinase enzyme preparation (mainly polygalacturonase activity but also pectin lyase, pectin-methyl-esterase and β-glucanase and protease as side activities) limited the polyphenol-cell wall interaction when enzyme, tannin and cell walls were added together, simultaneously [50]. This was possibly due to the enzymatic depectination of the cell walls and similar results were seen in apple tissue where depectination was the result of processing steps [64]. Bautista-Ortin et al. [77] used pure enzymes, namely a polygalacturonase, a cellulase and a commercial enzyme and achieved the same results. Interestingly the cellulase enzyme was the most effective. This was in contrast with earlier results from this group [66] where a decrease in pectin due to maceration enzyme action did not influence the capacity of Monastrell grapes to adsorb proanthocyanidins. Baustista-Ortin speculated that this was due to an increase in cell wall porosity which enhances encapsulation of proanthocyanidins.

It does seem that the extent of enzymatic degradation might be a very important factor determining the degree of proanthocyanidin absorption to the grape cell wall. Moderate cell wall degradation might increase the pores in the cell wall which could encapsulate and retain tannins. More extensive degradation can liberate high concentrations of both proanthocyanidins as well as cell wall degradation products (e.g., cell wall proteins) which can interact [77] and result in precipitation and removal during fining. In contrast there is evidence [85,86] that proanthocyanin-polysaccharide complexes form loosely packed oligomers or micro-gels that remain suspended. This might imply that these complexes will end up in the final wine. Finally, extreme enzymatic hydrolysis might reduce cell wall oligosaccharides to very small polymers, which have low affinity for tannins due to a low number of reactive binding sites; these released tannins may interact with protein and form precipitation as mentioned previously. For future research it might be important to determine what type of polyphenol complexes (whether it is polyphenol interactions with each other or with cell wall components) are important for sensory attributes such as astringency. Moreover in order to enhance favourable wine characteristics (such as mouthfeel, colour etc.) it is not clear what type of maceration and ageing methods are effective in this regard. It would be interesting to know for example what conformations of grape cell wall derived oligosaccharides and polysaccharides stabilise polyphenols during the crush and vinification stages. Another important aspect would be to know if the human gut successfully absorbs these polyphenol-polysaccharide colloidal aggregates during wine consumption. 

## 6. Developing Models of Wine Grape Berry Cell Wall Architecture from Enzyme and Glyco-Array Approaches 

Several hypothesis have been proposed over the last forty years to develop a unified model for plant cell walls; the first being Keegstra et al. [87]; based on biochemical methods of the time. Since then other models have been put forth; for example Carpita and Gibeaut [88] emphasizing the differences between monocotyledons and dicotyledons in their relative leaf cell wall compositions and general architecture. It is for example more common in dicotyledonous plants such as grapevines to have equal proportions of cellulose, hemicellulose (e.g., xyloglucans) and pectins in their primary cell walls. This is different from monocotyledonous plants where large amounts of secondary cell walls are present (e.g., xlyan-rich layers); thereby shifting this proportion based on dry weight sugar compositions as expounded in Carpita and Gibeaut [88]. The most popular model to date is the tethered-network model, which shows the cellulose microfibrils ‘tethered’ by xyloglucan strands through hydrogen bonding; this skeletal framework is then embedded in pectins, proteins and then other neutral polymers such as xylans. 

However a more realistic model of an Arabidopsis leaf cell wall is provided in Somerville et al. [4]. The model addressed still representing the basic ‘tethered-network model’ shows that the cell wall is a much more intermeshed structure that contains a number of distinct polymer types which act as building blocks (e.g., cellulose, xyloglucan, mannans, proteins and pectins). It is also important to note the degree of inter-polymeric and intra-polymeric (covalent and non-covalent) cross-linking (i.e., calcium egg-boxes, borate, side chain links, hydrogen bonding, oxidative bridges etc.) that occurs, giving rise to higher level architecture. The model also presents xylans and lignins being; present mostly in vascular tissue with secondary cell walls. In the case of grapes, being a fleshy berry fruit, these compounds would only be present in very low abundance in these tissues (e.g., in vascular strands and tissues). 

The gap is still present between knowing the chemical composition and the real architecture of berry cell wall. Understanding grape berry cell wall architecture is important as this matrix forms a barrier to extraction for polyphenols and other berry extractives that have to diffuse through these layers during wine maceration. For the last few decades, several analytical techniques have been implemented on grape berry cell wall and the most used methods are gas chromatography and size-exclusion chromatography [16,49,59,60]. The study of gene expression patterns of pathogens could also help us to infer the structure of specific plant tissue, it was shown that *Botrytis cinerea* highly expresses genes which encode the enzymes that target highly methyl-esterified HG, rhamnogalacturonan (RG), xyloglucan, xylan, mannan and cellulose [32], confirming previous studies on berry cell wall composition [5,16].

On the polymer level, the use of monoclonal antibodies (mAbs) and carbohydrate binding modules (CBMs) combined with high throughput microarray provides a fast and cost-effective way for grape and wine cell wall profiling [10,19]. This approach is termed comprehensive microarray polymer profiling (CoMPP). CoMPP is versatile in that new sets of mAbs and CBMs can be included or excluded providing a customizable profiling tool [89]. The mAbs and CBMs commonly used in our analyses are provided in Table 1. CoMPP can provide for high-throughput analysis, as the glycan microarray is able to print hundreds of samples (pectin-rich and hemicellulose-rich extracts from each grape cell wall sample) on one very small surface (e.g., a nitrocellulose membrane). Thus, hundreds of treatments of sample can be analyzed in parallel and together for comparative studies. After printing, the surface is probed with selected mAbs or CBMs (Table 1), respectively and then quantified by analyzing the antibody signals. Either the datasets acquired can be observed by creating a visual heatmap and/or raw-datasets can be used for multivariate data analysis.

**Figure 3:**
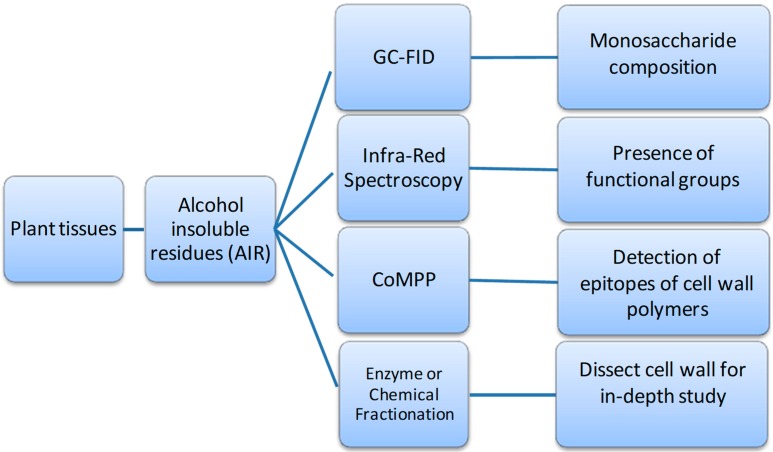
A combined approach using a series of cell wall analytical techniques in parallel, which provide a more comprehensive view of plant cell wall composition and structure.

However, each of these analytical chemistry-based techniques have their own set of advantages and disadvantages; the use of a combination of techniques can provide the data from different angles to generate a more complete description of the cell wall compositional and structural information. A work-flow was developed (which can be optimized for high-throughput analysis) for analyzing the same cell wall material (CWM) or alcohol insoluble residue (AIR) samples (see Figure 3) from a set of experiments which are divided into multiple directions (four in this case) for specific analytical techniques. These being: (1) gas chromatography methods for sugar composition after acid hydrolysis [90]; (2) ATR-FT-MIR (infrared) spectroscopy for functional group chemistry [91,92]; (3) Comprehensive Microarray Polymer Profiling (CoMPP) for polymer epitope abundance [10] and (4) mass spectrometry analysis after degradative techniques (usually enzymatic) [93]. Thereafter the datasets generated need to be analysed using various univariate and multivariate statistical approaches [21] to seek for significance and possible correlations.

This combined approach has been recently applied in a number of studies on wine maceration, and have generated several interesting datasets and outcomes. Firstly, the main cell wall polymers in grape berry pomace have been profiled and the distribution of these polymers were traced from berries into wine [28]; Secondly, the effect of ripening level on maceration efficacy, as well as the impact of commercial enzymes has also been investigated for a number of *Vitis vinifera* cultivars (i.e., Cabernet Sauvignon, Pinotage and Chardonnay) [19,94,95,96,97]. The most important benefit brought by CoMPP is that pectin and hemicellulose polysaccharide components from grape berries can be directly assessed using tissue/liquid extraction and probing techniques. By comparing the wine-soluble polysaccharides [28] or the pomace [95] or skin [96] cell walls before and after enzyme digestion, during winemaking (in vino) or in buffered model conditions, it was found that different individual enzyme(s) show varying impacts on the level of polysaccharide presence or absence.

**Figure 4:**
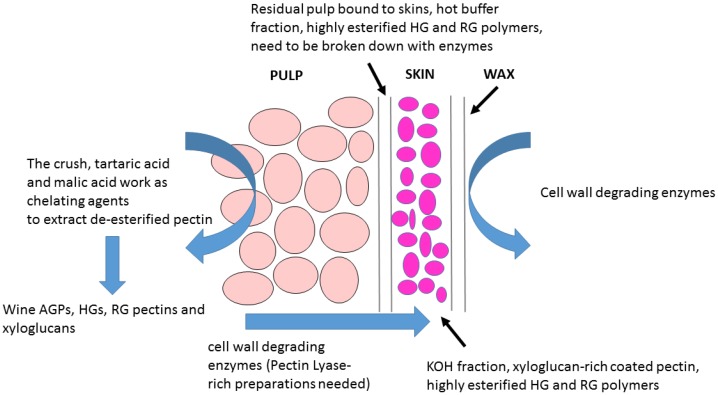
A simplified model of the wine grape cell wall showing the major tissue layers and the role of enzymes in maceration during fermentation.

The major key findings (see Figure 4) from using CoMPP in grape and wine studies thus far:

- A thorough study on Cabernet Sauvignon pectin polymers during winemaking yielded the first comprehensive wine grape berry cell wall model using recombinant enzymes. This new model replaces previous more ‘simplistic’ concepts in the literature [95]. Polygalacturonases and Pectinmethylesterases do NOT have a significant effect in Cabernet Sauvignon de-pectination/degradation—previous wine grape and enzyme models are therefore outdated [95]. RG-Lyases and Pectin Lyases are essential components for any commercial enzyme preparation to be effective in Cabernet Sauvignon winemaking releasing significant colour pigments and tannins into red wines [95].
- Overripe Pinotage grapes have a more de-pectinated cell wall composition and hence enzymes are more effective at early stages of ripening [96] and work in synergy [94]. Synergistic actions of purified enzymes help to achieve effective maceration, colour and tannin extraction and consistency of polyphenol levels in final Pinotage wines [19,95].
- Natural intra-vineyard wine grape cell wall variability occurs in a model Cabernet Sauvignon vineyard and effects colour and tannin extraction [19]. Commercial enzymes are able to reduce via de-pectination the natural intra-vineyard grape cell wall variability, which helps to achieve maceration that is more effective, colour and tannin extraction and consistency in wines from mixed harvests [19].
- Wine grape cell walls are composed of two major tissue layers (a) a pectin-rich tissue and (b) a pectin-coated hemicellulose-rich layer [28]. However, the RG-1-rich pectin coating-layer of Chardonnay wine grapes can be removed using a combination of hydrothermal pre-treatment and pectinase addition [97].
- A recent study on Shiraz grapes demonstrated a strong correlation between natural cell wall polysaccharide degradation caused by ripening and length of alcoholic fermentation with extraction of high molecular weight phenolics and tannins into wines [98,99].

In summary (see Figure 4) grapes have highly methylated complex layered cell walls, which require the action of RG-Lyases and Pectin-Lyases to breach the tissue during maceration and fermentation [95]. Enzyme preparations must be tested/validated for RG-Lyases and Pectin-Lyases for winemaking. The grape cell wall model developed (summarized in Figure 4) supports the design of new effective wine enzyme cocktails. However, limitations in knowledge still exist as more cultivars need to be tested, additional enzyme activities evaluated, the role of enzyme dosage is insufficiently studied, and wine sensory datasets should be assessed in future research investigations.

## 7. Conclusions

The global wine industry faces a number of challenges to satisfy consumers and remain commercially competitive, including (i) balancing improved juice yield with consistent wine quality, (ii) developing more effective maceration techniques and (iii) facilitating enhanced clarification (iv) while increasing the diversity of wine styles available on shelves. In addition to that, maintaining grape berry quality and yield in the face of climate change with the associated unpredictable biotic (e.g., disease pressures) and abiotic (e.g., water, light, heat variability) stressors requires new scientific tools and strategies to mitigate these impacts. The studies referenced in this review highlight the important role that the polysaccharide-rich berry cell wall plays in viticulture and oenology from the vine to the wine. Cell walls impact on berry integrity, the process of ripening, they act as the interface in plant protection (e.g., fungi) in the vineyard, mediate the release of chemical components (e.g., polyphenols) into wine and are the target polymers that enzymes act on during maceration and alcoholic fermentation in the cellar. 

Future research avenues naturally develop from the combination of our knowledge gaps in the literature and the new scientific resources that have become available in the last decade or so. The draft and updated genome (re-)sequences of grapevine with the large number of transcriptomic datasets generated for different grape developmental stages could be effectively combined with the high-throughput glyco-microarray (CoMPP) datasets and bioinformatics-chemometric analyses of cell wall metabolism. This strategy could facilitate in-depth understanding of (1) the role of cultivar specific differences in berry texture, resistance to biotic stressors and the associated phenolic extractability indexes during maceration. Further questions benefiting from glycoarray analyses include (2) studying the interaction between polyphenol and cell wall polymers in berries at different stages of ripeness. Hence the search for optimal ripeness for each cultivar could be impacted by cell wall conformational and architectural changes in combination with differential binding affinity of the polyphenol compounds present in vino. This may either positively or negatively affect extraction efficacy during winemaking and the nature of polysaccharide-polyphenol-protein precipitates found in wine tanks and bottles. Finally (3) the search for new enzyme sources (e.g., from metagenomics and microbiome studies) from vineyard and cellar environments holds the promise of discovering and producing novel enzyme cocktails. Glyco-array technology is an obvious screening tool that could identify/provide new improved alternatives to existing pectinases that would naturally be oenologically derived and safe for winemaking applications. The application of glycomics in grape growing and winemaking is opening new research avenues of significant relevance and importance to the global wine industry. 

## Figures and Tables

**Table 1:** The commonly used and characterised mAbs/CBMs for different groups of cell wall polymers.

Category	mAbs/CBMs	Epitope Recognition	Reference
HG	JIM5	HG with a low DE (mAb JIM5)	Clausen et al., 2003
	JIM7	HG with a high DE (mAb JIM7)	Clausen et al., 2003
	LM18	HG Partially methylesterified (mAb LM18)	Verhertbrugger et al., 2009
	LM19	HG Partially methylesterified (mAb LM19)	Verhertbrugger et al., 2009
	LM20	HG Partially methylesterified (mAb LM20)	Verhertbrugger et al., 2009
	2F4	HG Ca^2+^ crosslinked (mAb 2F4)	Ralet et al., 2010
	LM8	Xylogalacturonan (mAb LM8)	Ralet et al., 2010
RGI	INRA-RU1	Backbone of rhamnogalacturonan I (mAb INRA-RU1)	Ralet et al., 2010
	INRA-RU2	Backbone of rhamnogalacturonan I (mAb INRA-RU2)	
RGI side chains	LM5	(1→4)-β-d-galactan (mAb LM5)	Jones et al., 1997
	LM6	(1→5)-α-l-arabinan (mAb LM6)	Willats et al., 1998
	LM13	Linearised (1→5)-α-l-arabinan (mAb LM13)	Verhertbrugger et al., 2009
Mannan	LM21	(1→4)-β-d-(galacto)(gluco)mannan (mAb LM21)	Marcus et al., 2009
	LM22	(1→4)-β-d-(gluco)mannan (mAb LM22)	
Glucan, xyloglucan	BS-400-2	(1→3)-β-d-glucan (mAb BS-400-2)	Meikle et al., 1991
	LM15	Xyloglucan (XXXG motif) (mAb LM15)	Marcus et al., 2008
	LM25	Xyloglucan/unsibstituted β-d-glucan (mAb LM25)	Pedersen et al., 2012
Xylan/cellulose	LM10	(1→4)-β-d-xylan (mAb LM10)	McCartney et al., 2005
	LM11	(1→4)-β-d-xylan/arabinoxylan (mAb LM11)	
	CBM3a	Celulose (crystalline) (CBM3a)	Tormo et al., 1996
Extensins	LM1	Extensin (mAb LM1)	Smallwood et al., 1995
	JIM11	Extensin (mAb JIM11)	
	JIM20	Extensin (mAb JIM20)	
AGP	JIM8	AGP (mAb JIM8)	McCabe et al., 1997
	JIM13	AGP (mAb JIM13)	Knox et al., 1991
	LM14	AGP (mAb LM14)	Moller et al., 2008
	LM2	AGP, β-linked GlcA (mAb LM2)	Smallwood et al., 1996
